# The Synthesis of FeS and Investigation on Electrochemical Sensing Toward Neuroprotector

**DOI:** 10.7759/cureus.58709

**Published:** 2024-04-22

**Authors:** Michael Zacharia Mathew, Sherin Celshia, Muthamizh Selvamani, Vasugi Suresh, Mohammed Asif Hussein

**Affiliations:** 1 Department of Physiology, Saveetha Dental College and Hospitals, Saveetha Institute of Medical and Technical Sciences, Saveetha University, Chennai, IND

**Keywords:** fes, xrd, energy-dispersive x-ray spectroscopy (edx), hydrothermal, electrochemical, electrode, sem, scan rate effect, cyclic voltammetric, ascorbic acid

## Abstract

Background

Electrochemical sensing is a versatile field that uses electrochemistry concepts to detect and measure various substances. It finds applications in clinical diagnostics and environmental monitoring. Scientists are currently working on creating reliable electrochemical sensing devices that can accurately detect ascorbic acid. Iron sulfide (FeS) has emerged as a promising material for these sensors due to its excellent electrical conductivity, catalytic activity, and stability.

Materials and methods

The FeS nanoparticles were synthesized through the hydrothermal method of synthesis. The glassy carbon electrode (GCE) with a surface area of 0.071 cm^2 ^was modified with FeS before the working electrode was mechanically polished with 1 µm, 0.3 µm, and 0.05 µm alumina pastes for mirror finishing. Then it was subjected to ultrasonication in double distilled water for a few minutes to clean the surface of GCE. The FeS suspension was prepared by dispersing 5 mg of FeS in 10 mL of ethanol during 20 minutes of ultrasonic agitation then the GCE was coated with 10 μL of the suspension by drop coating method and dried in air.

Results

In this study, FeS nanoparticles were synthesized by the hydrothermal method of synthesis, and it was tested for their electrochemical sensing properties by various tests. Based on the field emission-scanning electron microscope (FE-SEM) analysis, scan rate effect test, cyclic voltammetric test, X-ray diffraction (XRD), and energy-dispersive X-ray (EDX) spectroscopy analysis done and results obtained, it was seen that the synthesized FeS nanoparticles are highly pure and have a crystalline structure. FeS has an even morphology. The synthesized particles also showed highly sensitive and specific sensing toward ascorbic acid when compared to unmodified 10.1 µA electrodes with a sensing value of 12.51 μA, thereby fulfilling the aim of this study.

Conclusion

Based on the outcomes of the diverse tests carried out, it is evident that the sample displayed a high crystalline nature as indicated by the XRD test. Additionally, the sample exhibited a uniform morphology, exceptional stability, and remarkable sensitivity. The developed FeS-based electrochemical sensor was found to be exceptionally pure and showed excellent performance, showcasing both high sensitivity and selectivity toward ascorbic acid.

## Introduction

Vitamin C, scientifically referred to as ascorbic acid, is a vital water-soluble compound that plays a crucial role in various physiological processes within the body. Being an essential nutrient, it cannot be synthesized by the body itself and must be obtained through dietary sources. Ascorbic acid, with its chemical formula C_6_H_8_O_6_, is a cyclic ester consisting of six carbon atoms arranged in a chain, with two of them forming a double bond. The remaining carbon atoms are connected to hydroxyl and hydrogen groups. This compound exists as a white, crystalline powder that readily dissolves in water. Its slightly tangy taste contributes to the acidic flavor found in citrus fruits like oranges, lemons, and grapefruits, which are renowned for their high vitamin C content. Additionally, ascorbic acid can also be found in other fruits and vegetables, such as strawberries, kiwis, broccoli, bell peppers, and various citrus fruits [1]. Biosensors are devices that respond to particular substances in a sample by utilizing electrochemical, optical, or other transducers along with a biological recognition system to convert the concentration of the substance into an electrical signal. They are commonly employed in bioprocessing, medical diagnostics, agriculture, and environmental monitoring [2].

Eukaryotes are the only known prokaryotes that can synthesize ascorbic acid. The key functions necessary for animals and plants are not needed in prokaryotes or can be replaced by other chemicals because they cannot synthesize ascorbate or require it [3-4]. A key turning point in the history of nutrition was the realization of the significance of ascorbic acid in avoiding scurvy, a condition marked by anemia, bleeding, gum disease, and weakness. In the 18th century, British naval physician James Lind made the first observations regarding the benefits of citrus fruits in preventing scurvy in sailors, which led to an understanding of the significance of vitamin C in maintaining human health [5]. Ascorbic acid serves several other crucial purposes in addition to being a necessary nutrient. It functions as an antioxidant and aids in preventing cellular harm from reactive oxygen species and free radicals. Additionally, it supports the renewal of other antioxidants, including vitamin E. The synthesis of collagen, which is essential for the development and maintenance of connective tissue, is also influenced by vitamin C [6]. An antioxidant reduces free radical generation and storage in tissues, thereby preventing tissue depletion [7].

Iron sulfide (FeS) nanoparticles are extremely minute, typically measuring 1 to 100 nm in diameter. The chemical composition of FeS nanoparticles is predominantly iron (Fe) and sulfur (S). The FeS compound has good conductive and magnetic properties [8]. Due to their potential broad range of applications in areas like energy storage, medicine, catalysis, and environmental remediation, nanoscale FeS particles have recently attracted significant research interest. For example, nanoscale FeS particles, like mackinawite, have demonstrated great promise as adsorbents and reductants for a variety of contaminants [9]. Nanoparticles, due to their distinct physicochemical characterization, hold greater significance than parent materials [10]. Metallic nanoparticles have shown significant practical promise and have seen rapid advancements, making them very viable for therapeutic applications, notably in the domains of diagnostics and imaging [11].

Expanding and increasing the range of possible applications for functional nanomaterials requires the development of easy-to-use, quick, and environmentally friendly synthetic techniques. A closed system employing water as the solvent may complete the reaction at a specific temperature and pressure, simulating the formation of crystals during the natural mineralization process. Water's physical characteristics, such as vapor pressure, density, surface tension, viscosity, and ionic product, will be significantly altered by hydrothermal conditions. The hydrothermal approach was first applied to the crystallization process in 1882. In addition to producing highly crystalline products with a limited size distribution, high purity, and low aggregation, it also notably reduces the reaction temperature of systems [12].

Through the interaction of the target analytes with electrodes in an electrochemical cell, electrochemical sensing can identify the analytes. A redox reaction occurs when the target analyte comes into contact with the electrode surface, changing the electrode's electrical characteristics [13]. A three-electrode setup made up of a working electrode, a reference electrode, and a counter electrode is used for electrochemical detection. The working electrode can be altered using various materials to recognize or concentrate metal ions in a particular way. Heavy metal ions can be detected by measuring changes in current, potential, capacitance, electrochemical impedance, or electrochemiluminescence that are brought on by their presence. These detection signals allow for the classification of electrochemical sensing into amperometric, potentiometric, and electrochemiluminescent techniques [14].

There have been many previous studies done on electrochemical sensing of ascorbic acid. However, few studies have employed FeS nanoparticles for this purpose. This research is done because of all the peculiarities mentioned earlier and characteristics of the FeS nanoparticle and the importance of ascorbic acid in the human body. There are many other methods to sense various compounds: polymerase chain reaction, nuclear magnetic resonance (NMR) spectroscopy, enzyme-linked immunosorbent assay, fluorescence spectroscopy, etc. However, all of these methods are costly, demand a large sample volume, and can take a lot of time. On the other hand, electrochemically sensing compounds (ascorbic acid in this case) can occur within a fraction of a second (with better and deeper studies), are cost-effective, and require only a small sample for testing.

Due to the significance of ascorbic acid in numerous biological processes and the demand for precise and sensitive detection methods, the inquiry into electrochemical sensing toward ascorbic acid utilizing FeS nanoparticles is noteworthy. FeS-based sensors have the potential to be more advantageous in terms of reliability, stability, and environmental friendliness, making them excellent candidates for real-world applications in environmental monitoring, food analysis, and healthcare. The analysis of ascorbic acid in the lab has been done now. The future study possibility is to obtain ascorbic acid samples from blood or other body fluids and then use those samples for testing and analysis. Ascorbic acid samples from foodstuffs can also be analyzed. The long-term stability and shelf life of FeS-based sensors can be another area of future research.

## Materials and methods

Materials

Iron nitrate, thiourea (NH_2_CSNH_2_), deionized water, ethanol, methanol, Teflon-lined stainless steel autoclave (The Chemours Company, Wilmington, USA), magnetic stirrer, muffle furnace, beaker, glassy carbon electrode (GCE), counter electrode (platinum wire), reference electrode (Ag/AgCl), alumina pastes, FeS suspension, and pipette.

Synthesis of FeS nanoflower

For the synthesis of FeS nanostructures, a Teflon-lined stainless steel autoclave with a 100 mL capacity will be used. All of the chemicals were of analytical grade and used without any further purification. In a typical procedure, 40 mM of Iron nitrate, 40 mM of thiourea (NH_2_CSNH_2_), 20 mL of deionized water, and 60 mL of ethanol were taken in a beaker. Then the mixture was stirred with a magnetic stirrer for 15 minutes and transferred to the autoclave. After that, the autoclave was sealed and heated at 200℃ for 12 hours. After cooling to room temperature naturally, the products were washed repeatedly with distilled water and methanol. Finally, the products were dried at room temperature for 12 hours to obtain powders. The synthesized sample will then be annealed in air at a temperature of 600℃ for two hours in a muffle furnace.

**Figure 1:**
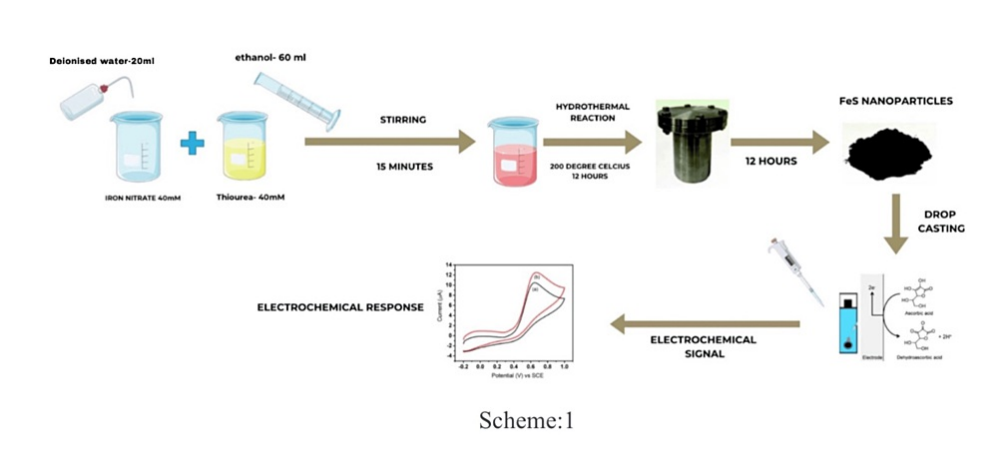
The process of synthesizing the required FeS nanoflower through the hydrothermal technique Image credit: Michael Zacharia Mathew and Muthamizh Selvamani FeS: ferrous sulfide; SCE: saturated calomel electrode

The process of synthesizing the required FeS nanoflower through the hydrothermal technique has been depicted in Figure 1. 

Electrode preparation procedure

The GCE was modified with FeS before that working electrode was mechanically polished with 1 µm, 0.3 µm, and 0.05 µm alumina pastes for mirror finishing. Then it was subjected to ultrasonication in double distilled water for a few minutes to clean the surface of GCE. The FeS suspension was prepared by dispersing 5 mg of FeS in 10 mL of ethanol during 20 minutes of ultrasonic agitation, Subsequently, the GCE was coated with 10 µL of the suspension using the drop coating method and then dried in air.

## Results

**Figure 2:**
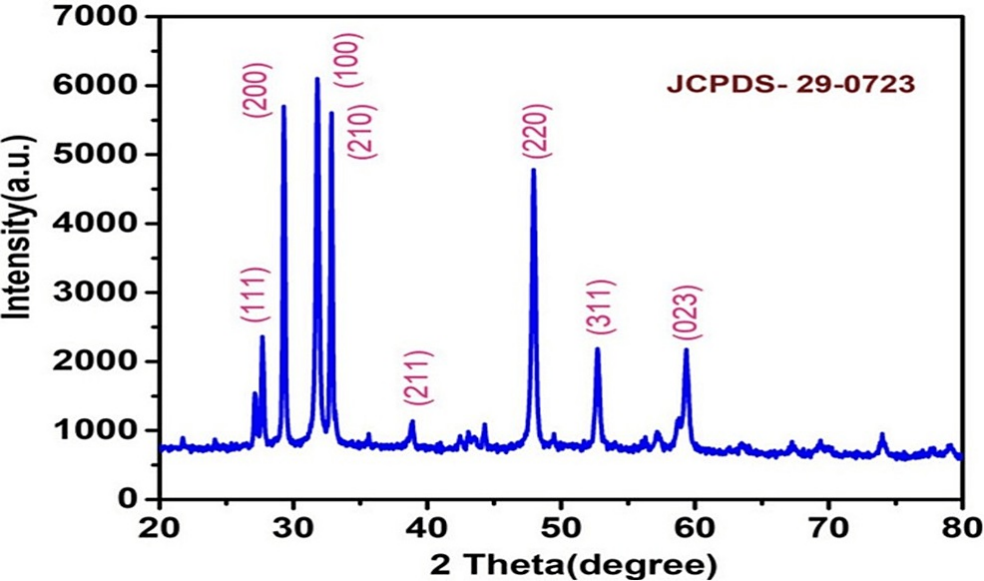
An XRD pattern of the electrochemically grown iron sulfide nanoflower after the hydrothermal treatment XRD: X-ray diffraction; JCPDS: Joint Committee on Powder Diffraction Standards

In materials science and crystallography, the atomic as well as the molecular structure of the crystalline material is investigated using X-ray diffraction (XRD) research. The structural nature of the prepared FeS sample is tested by employing the XRD analysis test. We have observed peaks with Miller indices of (111), (200), (100), (210), (211), (220), (311) and (023). The sharp peaks obtained from the analysis which can be seen in Figure 2 denote the highly crystalline structure of the prepared FeS compound with the Joint Committee on Powder Diffraction Standards (JCPDS)-29-0723.

Morphological analysis

Our ability to investigate the complex world of tiny structures has been completely transformed by the development of field emission-scanning electron microscopy (FE-SEM), a cutting-edge imaging method.

**Figure 3:**
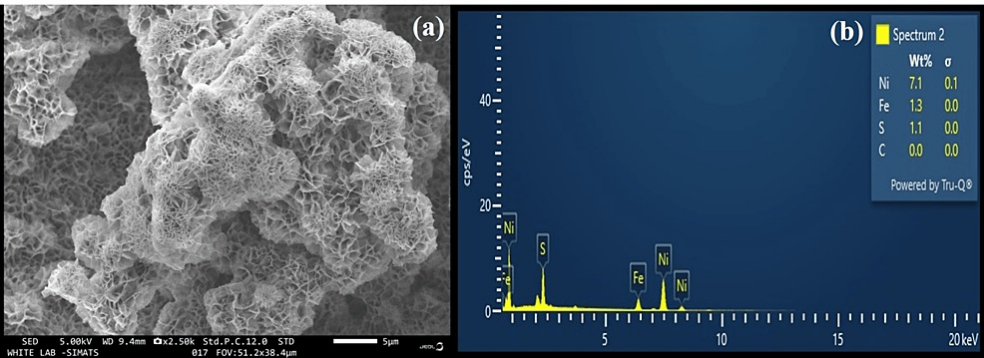
(a) The FE-SEM images of the FeS prepared by hydrothermal treatment. (b) The EDX analysis result of the FeS particles synthesized EDX: energy-dispersive X-ray; FE-SEM: field emission-scanning electron microscopy; FeS: ferrous sulfide

Figure 3a shows the FE-SEM images of the FeS prepared by hydrothermal treatment. Based on the analysis conducted and the results obtained, it can be seen that the compound is homogeneous and has an even morphology with a flower pattern. Due to its high sensitivity in detecting the various elements in tissues, energy-dispersive X-ray (EDX) microanalysis is used in numerous biomedical fields of study. Figure 3b shows the EDX analysis result of the FeS particles synthesized. The weight percentage of iron particles was 1.3 wt% and that of sulfur particles was 1.1 wt%. Nickel, comprising 7.1% of the total weight, results from the growth of the FeS substance on nickel foam. The EDX analysis test result shows the purity of the sample by exhibiting the absence of any impurities in the prepared FeS sample.

Cyclic voltammetric (CV) analysis

**Figure 4:**
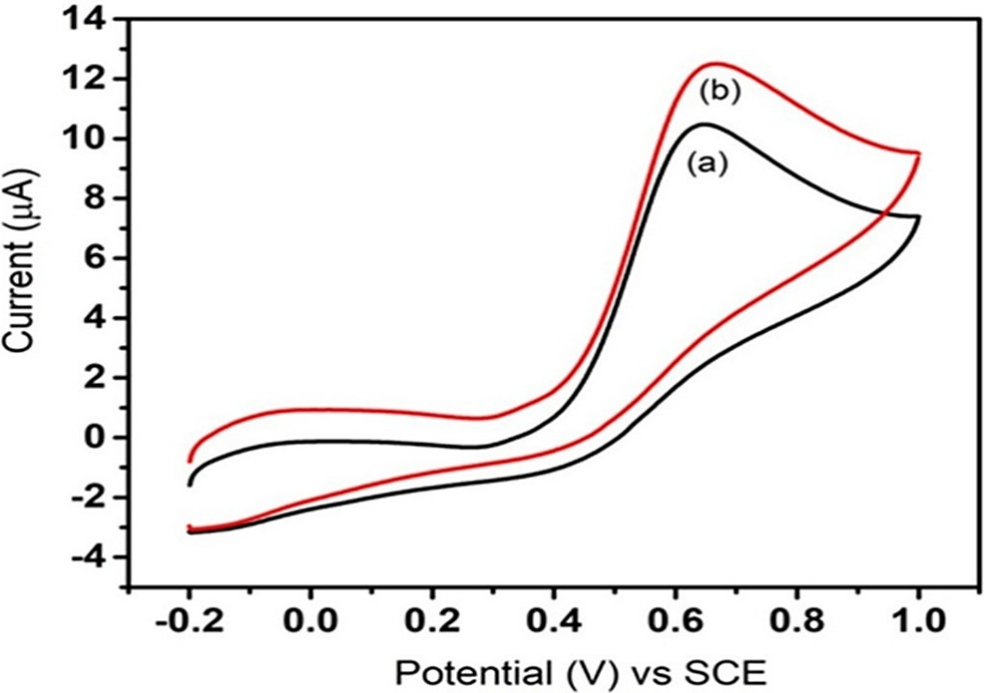
Cyclic voltammetric response of bare and FeS-modified GCE toward ascorbic acid at 50 mV/s GCE: glassy carbon electrode; SCE: saturated calomel electrode; FeS: ferrous sulfide

To investigate the redox behavior and electrochemical properties of FeS toward the detection of ascorbic acid, CV is employed in analytical chemistry and materials science. Figure 4 shows the CV test result of the FeS nanoflower that was tested. In Figure 4, the black line (a) denotes the current response of the standard GCE, and the red line (b) denotes the current response of the electrode modified with FeS nanoflower suspension in response to an applied potential of 50 mV/s.

**Figure 5:**
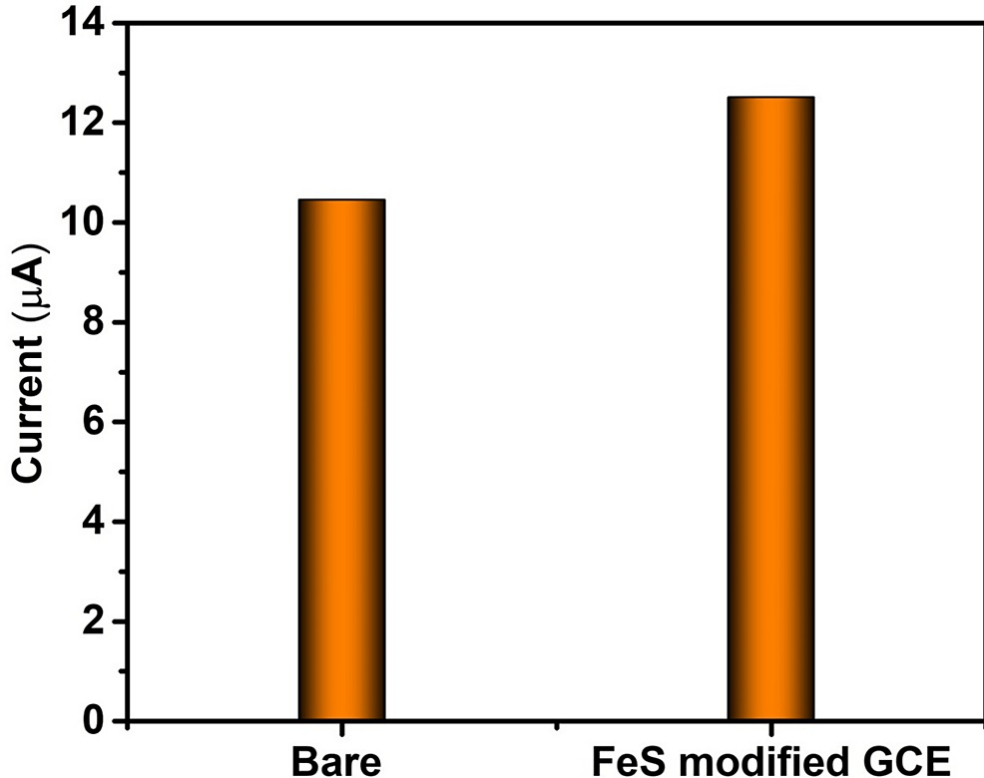
Cyclic voltammetry current response of bare and FeS-modified electrode toward ascorbic acid GCE: glassy carbon electrode; FeS: ferrous sulfide

In Figure 5, for the bare electrode, a potential of 50 mV/s resulted in a corresponding current response of 10.45 μA, while for the electrode modified with FeS nanoflower, the application of a potential of 50 mV/s led to a corresponding current response of 12.51 μA. The increased current response denotes the increased sensing ability of FeS nanoflower toward ascorbic acid.

Scan rate effect

**Figure 6:**
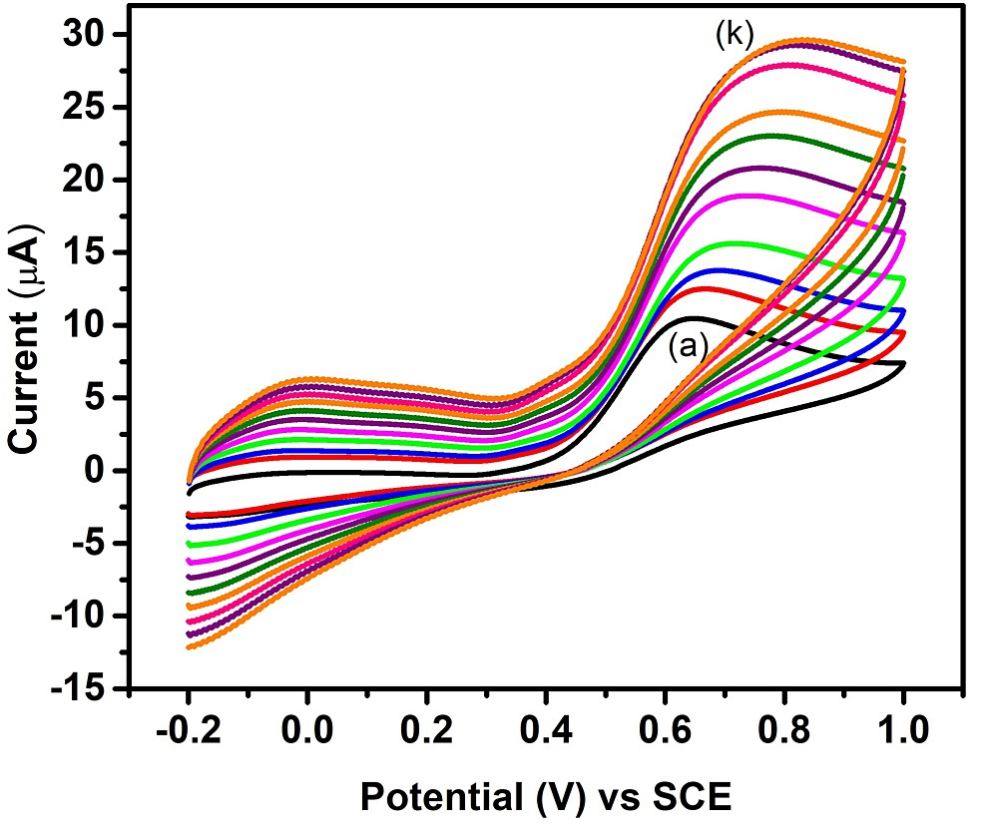
Scan rate effect of FeS-modified GCE toward ascorbic acid 10-110 mV GCE: glassy carbon electrode; FeS: ferrous sulfide; SCE: saturated calomel electrode; (a-k): 10-110 mV

The process involves gradually changing the voltage at various scan rates (10-110 mV), which produces diverse modifications in the resulting cyclic voltammograms. Figure 6 shows the scan rate effect test analysis result for FeS nanoflower synthesized. The current response readings were recorded while applying potentials ranging from 10 mV/s to 110 mV/s, with increments of 10 mV/s each time, as shown in Figure 6. As observed from the results and graphs plotted, it can be assessed that there was a subsequent increase in the current response in accordance with the increasing potentials applied subsequently one after the other. This finding confirms the high stability as well as the sensitivity of the FeS nanoflower toward the sensing of ascorbic acid.

## Discussion

The production of FeS nanoparticles and their use in ascorbic acid detection are important scientific fields, especially those related to nanotechnology and electrochemical sensing. FeS nanostructures must be carefully fabricated to modify their characteristics, maximize their electrochemical performance, and make them more appropriate for particular uses. An important antioxidant and staple of the human diet, ascorbic acid is crucial for preserving physiological health [15]. Nonetheless, high or low concentrations can have significant negative effects on health, thus accurate and trustworthy detection is essential. Because of their high surface area and electrocatalytic activity, FeS nanoparticles are a unique material that can be used as a sensing material for ascorbic acid.

In materials science and crystallography, the atomic as well as the molecular structure of the crystalline material is investigated using XRD research. It operates by exposing the crystalline sample to X-rays, which interact with the crystal lattice and scatter in various directions as a result. Scientists may measure the distances between atoms in the crystal lattice by analyzing the angles and intensities of these dispersed X-rays, which enables them to establish the crystal structure of the compound, phase composition, as well as other properties of the substance. It aids in understanding the composition and characteristics of crystalline substances [16].

Figure 1 shows an XRD pattern of the electrochemically grown FeS nanoparticle after the hydrothermal treatment. The composition and characteristics of the synthesized FeS compound were tested and confirmed by the XRD analysis. The results indicate the absence of peaks corresponding to any substances other than FeS. The obtained peaks were then compared with that of the standard JCPDS no: 29-0723 and were found to match. We have observed peaks with Miller indices of (111), (200), (100), (210), (211), (220), (311), and (023). The tall peaks obtained from the analysis which can be seen in Figure 2 denote the highly crystalline structure of the prepared FeS compound.

Our ability to investigate the complex world of tiny structures has been completely transformed by the development of FE-SEM, a cutting-edge imaging method. FE-SEM uses a focussed electron beam instead of an optical lens to examine the surfaces of various samples, as opposed to traditional optical microscopes. The sample is then ready for introduction into the FE-SEM chamber, where fine instrument modifications are made to improve imaging conditions such as beam energy and focus. The electron beam scans the sample's surface repeatedly in a grid pattern while imaging is being done. Backscattered and secondary electrons are produced as a result of interactions between electrons and the material and they are collected and converted into precise pictures. These pictures show the surface of the compound's intricacies in terms of morphology, topography, and structural elements [17].

Figure 3a shows the FE-SEM images of the sample prepared from hydrothermal treatment. Based on the analysis conducted and the results obtained, it can be seen that the compound is homogeneous and has an even morphology. It can also be seen that the prepared FeS nanoparticle has a characteristic flower-like pattern. FeS nanoflowers typically consist of hexagonal mackinawite (FeS) phases. These nanoflowers are composed of FeS nanoparticles arranged in a unique flower-like morphology. The FE-SEM image scale indicates that the particles are very small and the thickness of individual layer-like structures seen in the diagram are of very small nanometers. Hence, it has a small size as well.

Due to its high sensitivity in detecting the various elements in tissues, EDX microanalysis is used in numerous biomedical fields of study. The foundation of the elemental analysis method known as EDX microanalysis is the creation of distinctive X-rays in the specimen's atoms by the incident beam electrons. The application of EDX techniques in scientific research to examine the properties of elemental mapping, and topographical microstructure has grown over time [18-19].

Figure 3b shows the EDX analysis result of the FeS particles synthesized. Based on the EDX analysis done and the results obtained from the EDX analysis, it can be seen that the synthesized FeS nanoparticles are composed only of iron and sulfur particles. No other impurities or other particles were present. The presence of nickel particles is seen only because these FeS nanoparticles were grown on nickel foam. The weight percentage of iron particles was 1.3 wt% and that of sulfur particles was 1.1 wt%. The weight percentage of nickel was 7.1 wt%. The EDX analysis test result shows the purity of the sample by exhibiting the absence of any impurities in the prepared FeS sample.

Hence, it can be assessed that the prepared FeS sample is highly pure. To investigate the redox behavior and electrochemical properties of substances, CV, a potent electrochemical technique, is frequently employed in analytical chemistry and materials science. A controlled voltage or potential is applied to an electrochemical cell during the CV process, and the resulting current is then measured. A cyclic voltammogram can provide details about a substance's redox potential, electrochemical kinetics, etc. by graphing the current as a function of voltage [20-21]. Figure 4 shows the CV test result of the FeS nanoparticle that was tested. Based on the test conducted and the results obtained, it can be seen that when a potential of 50 mV was applied, a corresponding current response was observed. It can be seen that for the same potential of 50 mV applied to both the standard GCE as well as the electrode coated with FeS nanoparticle suspension (through the drop-casting process), the current responses of the respective electrodes were different. In Figure 4, the black line (a) denotes the current response of the standard GCE, and the red line (b) denotes the current response of the electrode coated with FeS suspension in response to an applied potential of 50 mV. From the results obtained, it can be seen that the modified electrode gives a higher current response to the applied potential of 50 mV when compared to that of the standard GCE. In Figure 5, for the bare electrode, applying a potential of 0.6 V resulted in a corresponding current response of 10.1 μA, whereas for the electrode modified with FeS nanoparticles, applying a potential of 0.621 V led to a corresponding current response of 12.1 μA. The increased current response denotes the increased sensing ability of FeS nanoparticles toward ascorbic acid.

The process of performing scan rate effect tests involves gradually changing a voltage at various scan rates, which produces diverse modifications in the resulting cyclic voltammograms. The scan rate effect is a crucial tool for comprehending the dynamic behavior of electrochemical systems because it provides important insights into the rate constants, diffusion coefficients, etc. of the compounds involved [22].

Figure 6 shows the scan rate effect test analysis result for the FeS nanoparticles synthesized. In this analysis, a particular potential is applied and the corresponding current response is recorded. Based on the conducted tests and the plotted graph, the black line shows the current response exhibited by the electrode modified with FeS nanoparticles in response to an applied potential of 10 mV. Following this a particular potential was again applied, and the corresponding current response was recorded. This process was repeated 10 more times and the applied potential was increased in increments of 10 mV each time. The current response readings were recorded from an applied potential of 10 mV to 110 mV, with increments of 10 mV each time. As observed from the results and plotted graph, it can be assessed that there was a subsequent increase in the current response in accordance with the increasing potentials applied subsequently one after the other. This finding confirms the high stability as well as the sensitivity of the FeS nanoparticle toward the sensing of ascorbic acid. It demonstrates the stability of the catalyst on the surface of the electrode.

Some similar previous studies have been conducted. In one such study, lead sulfide nanoparticles were synthesized by hydrothermal method, and then iron-doped lead sulfide nanoparticles were used for the electrochemical detection of chloramphenicol, an antibiotic [23]. The results indicated that iron-doped lead sulfide can be used as a good drug sensor. The results of our study showed that FeS nanoparticles can be used as a good sensor of ascorbic acid. In another study, a pyrolytic carbon film electrode was fabricated through a chemical vapor deposition process. It was then employed in the electrochemical sensing toward ascorbic acid. In our study, we have used a hydrothermal method of synthesizing the nanoparticle [24]. In this particular study, the author used a linear sweep voltammetry test to analyze the sensing characteristic of ascorbic acid. In our study, we have used a CV test to analyze the sensing ability of the FeS nanoparticle toward ascorbic acid. The technique was effective and showed that the nanoflower had good sensing ability toward ascorbic acid.

In another study, an ionic liquid-based nanocomposite was used for the electrochemical sensing of ascorbic acid [25]. CV analysis was used to check its ability to sense ascorbic acid present in foodstuffs and pharmaceuticals, and the results show that it is a good sensor of ascorbic acid. In our study, we used FeS nanoparticles to sense the ascorbic acid that is readily available in the market (unlike the natural ascorbic acid present in foodstuffs that were sensed in the other study). 

## Conclusions

The culmination of various tests and analyses conducted on the sample has provided valuable insights into its properties and potential applications. Notably, XRD testing affirmed the sample's high crystallinity, suggesting a well-ordered atomic structure. The sample had even morphology high stability and high sensitivity. The extraordinary stability and sensitivity displayed by the manufactured FeS-based electrochemical sensor was one of the most encouraging findings. Its remarkable selectivity for ascorbic acid, combined with its high purity and reliable performance, makes it an important instrument in the field of electrochemical sensing. The fabricated FeS-based electrochemical sensor was purely high and exhibited promising performance, demonstrating good sensitivity and selectivity toward ascorbic acid. The laboratory analysis of ascorbic acid has been completed. The future study is to obtain ascorbic acid samples from blood or other body fluids and then use those samples for testing and analysis. Ascorbic acid samples from foodstuffs can also be analyzed. The long-term stability and shelf life of FeS-based sensors can be another area of future research. This study covers the practical importance of creating effective ascorbic acid sensors in addition to advancing the production of nanomaterials, with possible applications spanning from food analysis to healthcare.
